# Rapid detection of abrin in foods with an up-converting phosphor technology-based lateral flow assay

**DOI:** 10.1038/srep34926

**Published:** 2016-10-05

**Authors:** Xiao Liu, Yong Zhao, Chongyun Sun, Xiaochen Wang, Xinrui Wang, Pingping Zhang, Jingfu Qiu, Ruifu Yang, Lei Zhou

**Affiliations:** 1 Chongqing Entry Exit Inspection and Quarantine Bureau, Chongqing 400020, P. R. China; 2 Laboratory of Analytical Microbiology, State Key Laboratory of Pathogen and Biosecurity, Beijing Institute of Microbiology and Epidemiology, Beijing 100071, P. R. China; 3 Beijing Key Laboratory of POCT for Bioemergency and Clinic (No. BZ0329), Beijing 100071, P. R. China; 4 Department of Clinical Laboratory, Chinese People’s Liberation Army General Hospital, Beijing 100853, P. R. China; 5 College of Animal Science and Technology, Jilin Agricultural University, Changchun 130118, P. R. China; 6 Institute for Plague Prevention and Control of Hebei Province, Zhangjiakou 075000, P. R. China; 7 School of Public Health and Management, Chongqing Medical University, Chongqing 400016, P. R. China

## Abstract

Abrin is a natural plant toxin found in the seeds of *Abrus precatorius*. It may be used for food poisoning or bioterrorism, seriously endangering public health. In this study, a reliable method for the rapid detection of abrin in foods was developed, based on an up-converting phosphor technology-based lateral flow assay (abrin-UPT-LFA). Nine high-affinity monoclonal antibodies (mAbs) against abrin were prepared, and the optimum mAbs (mAb-6F4 and mAb-10E11) were selected for use in the assay in double-antibody-sandwich mode. The assay was confirmed to be specific for abrin, with a detection sensitivity of 0.1 ng mL^−1^ for standard abrin solutions. Good linearity was observed for abrin quantitation from 0.1 to 1000 ng mL^−1^ (*r* = 0.9983). During the analysis of various abrin-spiked food samples, the assay showed strong sample tolerance and a satisfactory limit of detection for abrin (0.5–10 ng g^−1^ for solid and powdered samples; 0.30–0.43 ng mL^−1^ for liquid samples). The analysis of suspected food samples, from sample treatment to result feed-back, could be completed by non-professionals within 20 min. Therefore, the abrin-UPT-LFA is a rapid, sensitive, and reliable method for the on-site detection of abrin in foods.

Abrin is found in the seeds of *Abrus precatorius*, and is one of the most toxic plant proteins. It is a heterodimeric glycoprotein, consisting of an A chain and a B chain^1^,^2^,^3^. The B chain facilitates the transfer of the toxin to eukaryotic cells, and the A chain then irreversibly inactivates ribosomes, leading to cell apoptosis and cell death. Because of its high toxicity to cells, abrin has been studied for use in cancer therapy^4^,^5^. However, it may also cause food poisoning and can potentially to be used as a bioterrorism agent because it is readily available and easily isolated from the seeds of *A. precatorius* or associated products (jewelry and rosary beads)^6^,^7^. Once ingested by a human, it commonly causes gastroenteritis, or dehydration and death in more severe cases. The reported 50% lethal oral dose (LD_50_) of abrin in humans is 3–7 μg kg^−1^ body mass, which is much lower than that of ricin (1 mg kg^−1^ body mass)^8^. There is still no specific antidote to abrin, although the symptoms of poisoning can be eased with supportive care^9^. Therefore, methods for the rapid and early detection of abrin are essential to prevent exposure to it and to initiate an emergency response to food poisoning and bioterrorism.

Among the currently used detection methods for abrin, antibody-based enzyme-linked immunosorbent assays (ELISAs) remain the standard technique, and are widely used to detect abrin in environmental and food samples. The reported limit of detections (LODs) for abrin with ELISAs are 0.1–0.5 ng mL^−1^ in buffer and 0.5–10 ng mL^−1^ in most food matrices^10^,^11^,^12^. However, this method involves several washing and incubation steps, which take time (2–3 h), require professional skill, and even pose a risk of the dispersal of contaminants. Many other methods have also been developed for the more sensitive detection of abrin, including chromatographic methods^13^,^14^, electrochemiluminescence^10^, aptamer-based assays^15^,^16^, and real-time PCR (targeting the abrin DNA)^17^. Nevertheless, most of these methods ask for high requirements of analytical instruments or operations, thus limiting rapidness and simplicity of their use in the field.

The colloidal gold-based lateral flow assay (LFA), provides a very simple, rapid, and cost-effective method for the on-site detection of abrin^18^,^19^,^20^. Qualitative or semi-quantitative results can be easily obtained with this assay in 10–15 min, but the LOD is not ideal, at 3–10 ng mL^−1^ in buffer. Although the sensitivity can be improved to a level similar to that of the ELISA (0.1 ng mL^−1^) using silver enhancement technology^18^, it reduces the user-friendliness of the method (needs additional treatment of a silver-nitrate-saturated pad and a reducer pad, and takes another 10 min). Compared with the colloidal gold-based LFAs, the up-converting phosphor technology-based lateral flow assay (UPT-LFA) can more sensitively and accurately quantitatively detect compounds in real samples^21^. UPT-LFA is a new emerging type of LFAs in the last two decades, which uses up-converting phosphors (UCPs) instead of colloidal gold as the reporter. Up-converting phosphors (UCPs) are lanthanide-doped crystal nanoparticles with a unique up-converting phenomenon (they emit visible light when excited by infrared light^22^). Thus, there is little environmental interference to the detecting signals when they are used as the reporter in LFAs. Studies have demonstrated the usefulness of UCP reporters in LFAs, and the assay sensitivity can be improved 10–100-fold compared with that of colloidal gold^21^. Moreover, UPT-LFAs can achieve quantitative detections through reading optical signals of the UCP reporters. UPT-LFAs also show strong sample tolerance for various food matrices and environmental samples when they are used for the on-site detection of various infectious pathogens and foodborne pathogens^23^,^24^,^25^.

In this study, high-affinity monoclonal antibodies (mAbs) directly against abrin were prepared and used to develop an UPT-LFA (abrin-UPT-LFA) for the rapid detection of abrin. The performance of the assay was comprehensively evaluated with standard abrin samples and simulated contaminated food samples. The results show that the assay is sufficiently rapid, sensitive, and specific, and can quantify abrin in a wide concentration range. The assay also presents strong sample tolerance for different foodstuffs, demonstrating its suitability for the rapid on-site detection of abrin.

## Results and Discussion

### Preparation and screening of mAbs against abrin

**Figure 1:**
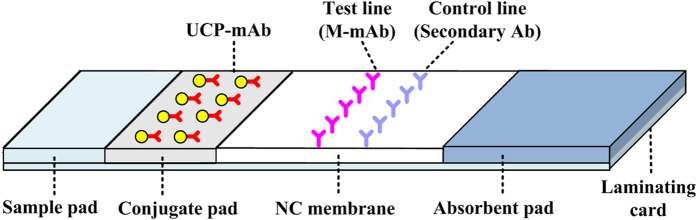
Schematic description of the abrin-UPT-LFA strip. The strip is typically composed of a sample pad, a conjugate pad, a NC membrane, an absorbent pad, and a laminating card. UCP-mAb conjugates were immobilized in the conjugate pad. Another mAb against abrin and the secondary antibody (goat anti-mouse IgG antibody) were coated on the NC membrane as the test line and control line, respectively.

In this study, nine specific mAbs against abrin were prepared with fine quantities and ELISA titers (see Supplementary Table S1). The mAbs were then conjugated to UCPs (UCP-mAb) applied in the conjugate pad, or were coated onto the nitrocellulose (NC) membrane as the test line (M-mAb) (Fig. 1). To identify the optimum mAb pair for the double-antibody-sandwich-based assay, the affinities for abrin of different mAb pairs were compared systematically, by estimating the initial LODs (without optimization) for abrin through abrin-UPT-LFA strips prepared with different mAb pairs (see Supplementary Table S2). During the test, the signal intensities for the test line and the control line are defined as the T value and C value, respectively; the ratio between the T value and C value (T/C) is the measurement result. Here, samples with T/C values higher than that of the blank control (phosphate buffer, PB) were roughly regarded as positive.

**Figure 2:**
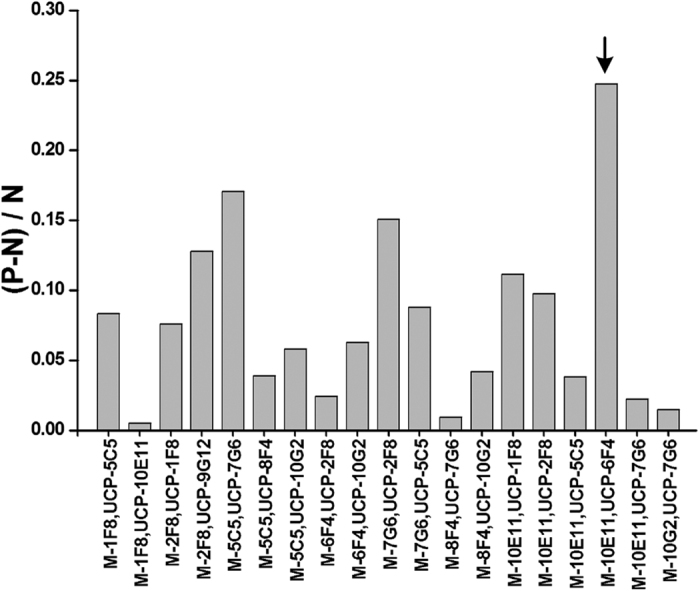
Comparison of the affinities of different mAb pairs for abrin (10 ng mL^−1^). The mAb pair M-10E11 and UCP-6F4, with the highest value for (P − N)/N, was selected as the optimum mAbs use in the abrin-UPT-LFA.

In total, 19 mAb pairs were selected as having lower initial LODs for abrin (10 ng mL^−1^) than the other pairs (≥100 ng mL^−1^) (Fig. 2). Among these mAb pairs, M-10E11 and UCP-6F4 showed a higher value for (P − N)/N (a measure of the affinity for abrin; “P” refers to a positive T/C value for abrin at a concentration of 10 ng mL^−1^; “N” refers to the T/C value of the blank.) than the other pairs. Consequently, the mAb pair (M-10E11 and UCP-6F4) was selected as the optimum antibodies for use in the abrin-UPT-LFA.

### Sensitivity, linearity, and precision of the abrin-UPT-LFA

**Figure 3:**
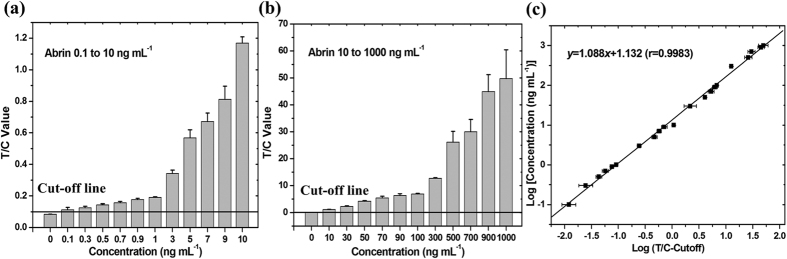
Detection of standard abrin samples of 0.1–10 ng mL^−1^ (**a**) and of 10–1000 ng mL^−1^ (**b**), and the quantification curve for abrin in the assay (**c**). On the curve, the *x*-axis refers to the logarithm of T/C –the cut-off value, and the *y*-axis refers to the logarithm of the abrin concentration (ng mL^−1^). Data are represented as mean ± s.d., n = 3.

After systematic optimizations, the performance of the assay was evaluated by testing a series of standard abrin solutions ranging in concentration from 0.1 to 1000 ng mL^−1^. The cut-off threshold for the assay was determined to be 0.1 (mean + 3SD of the T/C values) by testing the blank samples (PB); samples with T/C value higher than the cut-off value were determined as positive and vice versa. As shown in Fig. 3, as little as 0.1 ng mL^−1^ abrin could be positively detected, so this was defined as the detection sensitivity of the assay. The T/C value gradually increased as the abrin concentrations increased from 0.1 to 1000 ng mL^−1^, which provides the basis for abrin quantitation. A standard curve and the quantitative equation are shown in Fig. 3c, with the logarithm of the T/C–cut-off value as *x* and the logarithm of the abrin concentration as *y*. The correlation coefficient (*r*) of linear correlation was determined to be 0.9983, demonstrating good linearity for the quantification of abrin in a wide concentration range of 0.1–1000 ng mL^−1^. Except at very high abrin concentrations (500–1000 ng mL^−1^), the coefficients of variation (CVs) for the repeated tests of each sample were all less than 15% (1.9%–12.4%).

### Specificity of abrin-UPT-LFA

**Figure 4:**
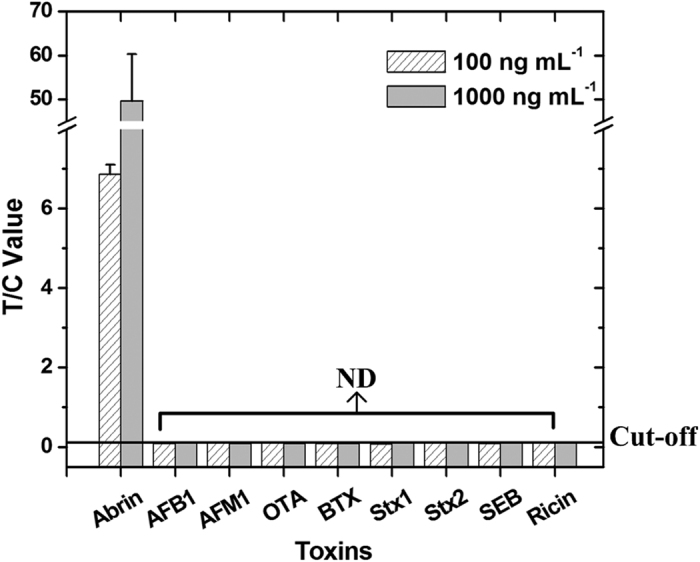
Specificity of the abrin-UPT-LFA. No cross-reactivity was detected with other toxins, even at high concentrations (100 ng mL^−1^ or 1000 ng mL^−1^). “ND” means “not detected”.

Toxins that have a similar structure or a similar contamination route to those of abrin or that could potentially be used as bioterrorism agents were selected to evaluate the specificity of the assay. These included ricin, aflatoxin B1 (AFB1), aflatoxin M1, ochratoxin A (OTA), botulinum toxin (BTX), shiga toxin 1 (Stx1), shiga toxin 2 (Stx2), and staphylococcal enterotoxin B (SEB). As shown in Fig. 4, none of these toxins were detected by the assay, even at high concentrations of 100 ng mL^−1^ and 1000 ng mL^−1^. These results demonstrate the high specificity of the assay for the detection of abrin.

### Detection of food samples spiked with abrin

**Figure 5:**
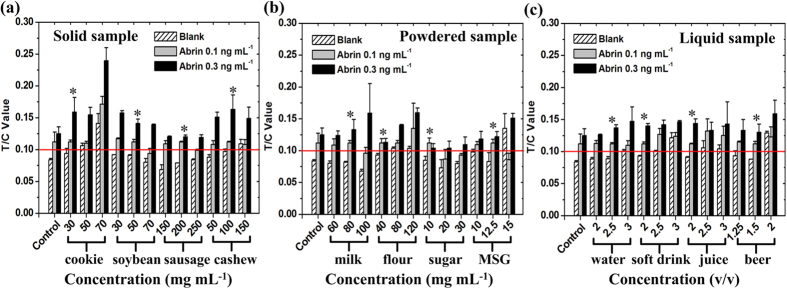
Performances of the abrin-UPT-LFA in detecting abrin in various spiked food samples. The *x*-axis refers to the different concentrations or volume ratios used for sample preparation, and the *y*-axis refers to the detection results (repeated three times) for abrin in samples using the abrin-UPT-LFA. Standard abrin solutions with no food matrices were used as the controls. *MST of each sample. Data are represented as mean ± s.d., n = 3.

The performance of the assay was also evaluated by testing different kinds of food samples spiked with abrin (0.1 or 0.3 ng mL^−1^), including solid samples, powdered samples, and liquid samples. Considering the likely effects of food matrices on the assay performances, we mixed a different weight or volume of each food sample with the sample treatment buffer (5 mL of PB; as shown in Fig. 5) for the sample preparation; then the simulated contaminated samples were tested with the assay. As the results show, within the proper concentration limit (marked with * in Fig. 5) for sample preparation, the sensitivity of the assay was maintained as 0.1 ng mL^−1^ for abrin in all kinds of foods. Beyond that limit, the food matrices affected the assay performance to different degrees. (1) The minimum concentration of abrin that could be detected increased to 0.3 ng mL^−1^ or higher when present in soybean (≥70 mg mL^−1^), sausage (≥250 mg mL^−1^), milk powder (≥100 mg mL^−1^), or sugar (≥20 mg mL^−1^). (2) False positive results were detected in cookies (≥50 mg mL^−1^), cashews (≥150 mg mL^−1^), flour (≥80 mg mL^−1^), monosodium glutamate (MSG) (≥15 mg mL^−1^), water (≥3:5), soft drink (≥2.5:5), juice (≥2.5:5), and beer (≥2:5).

The highest concentration (mg mL^−1^) or volume ratio (liquid sample volume/PB volume) used for sample preparation that did not reduce the assay performance (sensitivity and specificity) was determined to the maximum sample tolerance (MST). Table 1 presents the MSTs of the assay for various food samples and the corresponding LODs for abrin in the foods. For solid and powdered samples, the MSTs ranged from 10 to 200 mg mL^−1^, with corresponding LODs for abrin of 0.5–10 ng g^−1^. For liquid samples, the MSTs ranged from 1.5:5 to 2.5:5 (v/v), with LODs of 0.30–0.43 ng mL^−1^. The highest LOD for abrin was in the sugar samples, at 10 ng g^−1^. However, it still satisfied the clinically relevant level required to protect humans (The LD_50_ of abrin is 3–7 μg kg^−1^ body mass when ingested by humans. For a 50 kg adult, only when he consume at least 15 kg of sugar contaminated with abrin at a concentration of 10.0 ng g^−1^, the ingested abrin could reach the dose of LD_50_ [150 μg abrin]). These results demonstrate that the abrin-UPT-LFA has excellent sample tolerance and satisfied LODs for abrin in food samples.

## Conclusions

In this study, we developed the abrin-UPT-LFA for the rapid and reliable detection of abrin in food samples. The assay was shown to be highly sensitive (0.1 ng mL^−1^) and specific for abrin, attributable to the use of more sensitive reporter, UCPs, and the high-affinity anti-abrin mAbs prepared in this study. Abrin can also be quantitated in a wide concentration range of 0.1–1000 ng mL^−1^ (*r* = 0.9983). To detect abrin in foods, the assay does not require complex sample treatments and can be completed within about 20 min. And, this assay displays good sample tolerance to various foods (solids, powders, and liquids). The LODs were 0.5–10 ng g^−1^ and 0.30–0.43 ng mL^−1^ for abrin in solid/powdered and liquid samples, respectively, enabling the assay to detect trace amount of abrin contamination in food samples. In summary, the simplicity, robustness, and reliability of the proposed abrin-UPT-LFA make it suitable for the rapid on-site detection of abrin in foods.

## Materials and Methods

### Ethics statement

All animal experiments were carried out in accordance with the guidelines approved by the Committee for the Welfare and Ethics of Laboratory Animals, Beijing Institute of Microbiology and Epidemiology (Beijing, China). All experimental protocols were approved by the Ministry of Health in the General Logistics Department of the Chinese People’s Liberation Army (permit no. SCXK-2007-004). Eight-week-old female Balb/c mice were acquired from the Laboratory Animal Research Center, Academy of Military Medical Sciences (Beijing, China). The mice were housed in sterile isolator separately and provided with standard food and water. The health condition of the mice was monitored every day.

### Reagents and chemicals

UCPs (NaYF_4_:Yb^3+^, Er^3+^) with a diameter of about 50 nm were provided by Shanghai Kerune Phosphor Technology Co. Ltd (Shanghai, China). The excitation spectrum peak and emission spectrum peak of the UCPs were 980 nm and 541.5 nm, respectively. The NC membrane (SHF 1350225) and the glass fiber (GFCP 20300) were obtained from Millipore Corp. (Bedford, MA, USA). Papers (no. 470 and no. 903) were purchased from Schleicher & Schuell, Inc. (Keene, NH, USA). The laminating cards were purchased from Shanghai Liangxin Biotechnology Company (Shanghai, China). The plastic cartridge for the strip was designed by our group and manufactured by Shenzhen Jincanhua Industry Company (Shenzhen, China).

Bovine serum albumin V (BSA), polyethylene glycol 8000 (PEG 8000), Nonidet P 40 (NP-40), and casein were all of analytical grade, purchased from Sigma-Aldrich (St. Louis, MO, USA). Na_2_HPO_4_, KH_2_PO_4_, NaCl, HCl, NaOH, and other reagents were supplied by the Sinopharm Chemical Reagent Co., Ltd (Shanghai, China), and were used without further purification.

Abrin (identified as a mixture of abrin-a, -b, -c, -d, and agglutinin with mass spectrum and electrophoresis) and ricin were obtained from Beijing Hapten and Protein Biomedical Institute (Beijing, China). AFB1, AFM1, and OTA were purchased from Huaan Magnech Bio-Tech Co. Ltd (Beijing, China). Stx 1, stx 2, SEB, and BTX are preserved in our laboratory.

### Preparation and screening of mAbs against abrin

To prepare mAbs against abrin, Balb/c mice were immunized by the subcutaneous groin injection of inactivated abrin (treated with paraformaldehyde) every 2 weeks for 2 months. Then, the spleen cells of the mice were collected, suspended, and mixed with myeloma cells (SP2/0) to produce hybridomas. The hybridomas culture media were then screened with a direct ELISA (2 μg mL^−1^ abrin as the plate-coating antigen). The positive cell cultures were cloned with limiting dilution until the positive rate reach 100%. Ten days after the intraperitoneal injection of Balb/c mice with the positive hybridomas, the ascites containing the anti-abrin mAbs were collected and purified with the caprylic acid and saturated ammonium sulfate method. The titers and quantities of the mAbs were determined with direct ELISA and UV spectrometry, respectively.

### Preparation of the abrin-UPT-LFA strip

To prepare the abrin-UPT-LFA strip, several treatments were required. First, UCPs were coated with a layer of silica, and functionalized with amino- and aldehyde- groups; subsequently, the UCPs were covalently conjugated to the anti-abrin mAbs^26^,^27^. The conjugates (UCP–mAb, 1 mg mL^−1^) were then fixed onto the conjugate pad (glass fiber). Second, another anti-abrin mAb (2 mg mL^−1^) and the goat anti-mouse IgG polyclonal antibody (2 mg mL^−1^) were used to coat the NC membrane at 2 μL cm^−1^ as the test line and control line, respectively, with an IsoFlow Dispenser (Imagene Echnology, NH, USA). Third, the sample pad (no. 903 paper, 20 cm × 3 cm) was soaked in 3 mL of optimized buffer (0.03 M PB containing 1% BSA, 0.5% PEG 8000, 0.5% NP-40, and 0.25% casein, pH 7.2) for 1 min, and then dried at 37 °C for 2 h. Finally, the sample pad, conjugate pad, NC membrane, and absorbent paper (no. 470) were assembled together on the bottom of the laminating card and cut into 4 mm wide strips for later use.

### Development and optimization of abrin-UPT-LFA

Standard abrin solutions or abrin samples diluted with PB (100 μL) were directly applied to the abrin-UPT-LFA strip. After 15 min, the results were obtained with a UPT biosensor, which was designed and produced by our laboratory and Shanghai Institute of Optics and Fine Mechanics, Chinese Academy of Sciences (Shanghai, China). When excited by a laser of 980 nm, the optical signals generated by the UCPs on the membrane were collected with a photomultiplier tube (PMT) in the biosensor. And the PMT quantitatively transformed the optical signals to electric signals. The signal intensities from the test line and the control line are detected and quantified as the T value and C value, respectively. The ratio T/C is regarded as the measurement result, which can be used for further qualitative and quantitative analysis of the sample.

During the systematic optimization of the assay, different mAb pairs against abrin were compared to determine the optimum pair for use in the double-antibody-sandwich-based immunoassay. Standard abrin solutions (0, 10, and 100 ng mL^−1^ in PB) were tested with strips prepared with different mAb pairs. Samples with T/C values higher than that of the blank control (PB without abrin) were roughly regarded as positive. The mAb pair with the highest affinity for abrin [indicated by the lowest initial LOD or the highest (P − N)/N value] was selected as the optimum mAbs for the abrin-UPT-LFA.

### Evaluation of the sensitivity, linearity, and precision of the assay

The standard abrin solution (100 μg mL^−1^) was serially diluted 10-fold with PB to concentrations of 0.1–1000 ng mL^−1^. Each dilution (100 μL) was tested three times with the assay, as described above. The blank control (PB) was also tested to determine the cut-off threshold (mean + 3SD of the T/C values) of the assay. Abrin samples with a T/C value higher than the cut-off value were considered positive and vice versa. The lowest abrin concentration that could be positively detected was defined as the detection sensitivity of the assay. A standard quantification curve was plotted, with the logarithm of T/C – the cut-off on the *x*-axis and the logarithm of the abrin concentration on the *y*-axis. The correlation coefficient (*r*) was calculated to evaluate the linearity of the quantification curve. The CVs for the repeated measurements were determined to evaluate the precision of abrin quantitation with the assay.

### Evaluating the detection specificity of the assay

Various toxins were used to test the specificity of the assay: ricin, AFB1, AFM1, OTA, BTX, Stx1, Stx2, and SEB. Each toxin was diluted with PB to concentrations of 100–1000 ng mL^−1^, and then tested with the abrin-UPT-LFA as described above.

### Detection of abrin-spiked food samples

Different kinds of food samples, including solid samples (cookie, soybean, sausage, and cashews), powdered samples (milk powder, flour, white sugar, and MSG), and beverage samples (soft drink, juice, beer, and water), were obtained from local supermarkets. The samples were first homogenized; and a specific weight or volume of sample was added to 5 mL of PB (as shown in Fig. 5). Each sample matrix was then spiked with the standard abrin solution at a final concentration of 0.1 or 0.3 ng mL^−1^. After the mixture was thoroughly mixed and allowed to stand, 100 μL of the supernatant was introduced onto the strip and tested three times, as described above. Standard abrin solutions with no food matrix were used as the controls.

## Additional Information

**How to cite this article**: Liu, X. *et al.* Rapid detection of abrin in foods with an up-converting phosphor technology-based lateral flow assay. *Sci. Rep.* **6**, 34926; doi: 10.1038/srep34926 (2016).

## Supplementary Material

Supplementary Information

## Figures and Tables

**Table 1:** Assay sample tolerance and LODs of abrin in food samples.

Food Sample		MST	LOD* of abrin in food
Solid samples	Cookie	30 mg mL^−1^	3.33 ng g^−1^
	Soybean	50 mg mL^−1^	2.00 ng g^−1^
	Sausage	200 mg mL^−1^	0.50 ng g^−1^
	Cashew	100 mg mL^−1^	1.00 ng g^−1^
Powdered samples	Milk powder	80 mg mL^−1^	1.25 ng g^−1^
	Flour	40 mg mL^−1^	2.50 ng g^−1^
	Sugar	10 mg mL^−1^	10.0 ng g^−1^
	MSG	12.5 mg mL^−1^	8.00 ng g^−1^
Liquid samples	Water	2.5:5 (v/v)	0.30 ng mL^−1^
	Soft drink	2:5 (v/v)	0.35 ng mL^−1^
	Juice	2:5 (v/v)	0.35 ng mL^−1^
	Beer	1.5:5 (v/v)	0.43 ng mL^−1^

“v/v” refers to the volumetric ratio between the sample and the sample treatment solution (PB). For example, 2:5 (v/v) means that 2 mL of liquid sample was mixed into 5 mL of PB.

*For solid and powered samples, the LOD is calculated as “the detection sensitivity (0.1 ng mL^−1^) / the corresponding MST”; for liquid samples, the LOD is calculated as “the detection sensitivity (0.1 ng mL^−1^) × the sample dilution fold of the MST”.
