# Supplementary material for: Rapid detection of abrin in foods with an up-converting phosphor technology-based lateral flow assay
Source: Sci Rep. 2016 Oct 5;6:34926. doi: 10.1038/srep34926 (PMC5050493; doi:10.1038/srep34926)
Supplement: Supplementary Information [file srep34926-s1.pdf]

Supplementary Information

Rapid detection of abrin in foods with an up-converting phosphor technology-based lateral flow assay

Xiao Liu[1,2,3†], Yong Zhao[2,3†], Chongyun Sun[2,3,4], Xiaochen Wang[2,3,5], Xinrui Wang[2,3,6], Pingping Zhang[2,3], Jingfu Qiu[7]*, Ruifu Yang[2,3]* & Lei Zhou[2,3]*

[1]Chongqing Entry Exit Inspection and Quarantine Bureau, Chongqing 400020, P. R. China

[2]Laboratory of Analytical Microbiology, State Key Laboratory of Pathogen and Biosecurity, Beijing Institute of Microbiology and Epidemiology, Beijing 100071, P. R. China

[3]Beijing Key Laboratory of POCT for Bioemergency and Clinic, Beijing 100071, P. R. China

[4]Department of Clinical Laboratory, Chinese People's Liberation Army General Hospital, Beijing 100853, P. R. China

[5]College of Animal Science and Technology, Jilin Agricultural University, Changchun 130118, P. R. China

[6]Institute for Plague Prevention and Control of Hebei Province, Zhangjiakou 075000, P. R. China

[7]School of Public Health and Management, Chongqing Medical University, Chongqing 400016, P. R. China

[†] XL and YZ contributed equally to this work.

*Corresponding authors:

Lei Zhou, Tel: +86-10-66948562; E-mail: ammszhoulei@aliyun.com

Ruifu Yang, Tel: +86-10-66948595; E-mail: ruifuyang@gmail.com

Jingfu Qiu, E-mail: jfqiu@126.com

Supplementary Table S1. Quantities and titers of mAbs against abrin prepared in this study.

**mAb**	**1F8**	**2F8**	**5C5**	**6F4**	**7G6**	**8F4**	**9G12**	**10E11**	**10G2**
**Quantities (mg mL^{-1})**	10	8	18	10	13	18	8	7	11
**Titers (ng mL^{-1})**	15.6	15.6	15.6	31.25	15.6	125	31.25	31.25	31.25

Supplementary Table S2. Comparison of different mAb pairs for use in the double-antibody-sandwich-based abrin-UPT-LFA.

M-mAb	Initial LODs for abrin (ng mL^{-1}) with different UCP-mAb conjugates								
	**1F8**	**2F8**	**5C5**	**6F4**	**7G6**	**8F4**	**9G12**	**10E11**	**10G2**
**1F8**	-	F	**10**	100	100	F	100	**10**	100
**2F8**	**10**	-	F	100	F	F	**10**	F	F
**5C5**	F	100	-	F	**10**	**10**	F	100	**10**
**6F4**	F	**10**	F	-	F	F	F	100	**10**
**7G6**	F	**10**	**10**	100	-	100	100	F	F
**8F4**	100	F	F	F	**10**	-	F	100	**10**
**9G12**	F	F	F	100	F	F	-	F	F
**10E11**	**10**	**10**	**10**	**10**	**10**	100	F	-	100
**10G2**	F	F	100	100	**10**	100	F	100	-

"-" means "not used to prepare the strip". "F" means "failed to detect positive samples (10, 100 ng mL^{-1}) in the assay". The mAb pairs with lower initial LODs for abrin are shown in bold.
